# GB Virus C/Hepatitis G Virus Envelope Glycoprotein E2: Computational Molecular Features and Immunoinformatics Study

**DOI:** 10.5812/hepatmon.15342

**Published:** 2013-12-30

**Authors:** Mohammad Mahdi Ranjbar, Khodayar Ghorban, Seyed Moayed Alavian, Hossein Keyvani, Maryam Dadmanesh, Abbas Roayaei Ardakany, Mohammad Hassan Motedayen, Alireza Sazmand

**Affiliations:** 1 Department of Immunology, University of Tehran, Tehran, IR Iran; 2 Department of Immunology, School of Medicine, AJA University of Medical Sciences, Tehran, IR Iran; 3 Middle East Liver Diseases Center (MELD), Tehran, IR Iran; 4 Baqiyatallah Research Center for Gastroenterology and Liver Diseases, Baqiyatallh University of Medical Sciences, Tehran, IR Iran; 5 Department of Virology, Tehran University of Medical Sciences, Tehran, IR Iran; 6 Department of Infectious Diseases, School of Medicine, AJA University of Medical Sciences, Tehran, IR Iran; 7 Department of Computer Sciences, University of Nevada, Reno, USA; 8 Razi Vaccine and Serum Research Institute, Karaj Branch, Karaj, IR Iran; 9 Department of Agriculture, Payame Noor University, Yazd, IR Iran

**Keywords:** GB virus C, glycoprotein E2, GB virus C, Immunoinformatics

## Abstract

**Introduction:**

GB virus C (GBV-C) or hepatitis G virus (HGV) is an enveloped, RNA positive-stranded flavivirus-like particle. E2 envelope protein of GBV-C plays an important role in virus entry into the cytosol, genotyping and as a marker for diagnosing GBV-C infections. Also, there is discussion on relations between E2 protein and gp41 protein of HIV. The purposes of our study are to multi aspect molecular evaluation of GB virus C E2 protein from its characteristics, mutations, structures and antigenicity which would help to new directions for future researches.

**Evidence Acquisition:**

Briefly, steps followed here were; retrieving reference sequences of E2 protein, entropy plot evaluation for finding the mutational /conservative regions, analyzing potential Glycosylation, Phosphorylation and Palmitoylation sites, prediction of primary, secondary and tertiary structures, then amino acid distributions and transmembrane topology, prediction of T and B cell epitopes, and finally visualization of epitopes and variations regions in 3D structure.

**Results:**

Based on the entropy plot, 3 hypervariable regions (HVR) observed along E2 protein located in residues 133-135, 256-260 and 279-281. Analyzing primary structure of protein sequence revealed basic nature, instability, and low hydrophilicity of this protein. Transmembrane topology prediction showed that residues 257-270 presented outside, while residues 234- 256 and 271-293 were transmembrane regions. Just one N-glycosylation site, 5 potential phosphorylated peptides and two palmitoylation were found. Secondary structure revealed that this protein has 6 α-helix, 12 β-strand 17 Coil structures. Prediction of T-cell epitopes based on HLA-A*02:01 showed that epitope NH3-LLLDFVFVL-COOH is the best antigen icepitope. Comparative analysis for consensus B-cell epitopes regarding transmembrane topology, based on physico-chemical and machine learning approaches revealed that residue 231- 296 (NH2- EARLVPLILLLLWWWVNQLAVLGLPAVEAAVAGEVFAGPALSWCLGLPVVSMILGLANLVLYFRWL-COOH) is most effective and probable B cell epitope for E2 protein.

**Conclusions:**

The comprehensive analysis of a protein with important roles has never been easy, and in case of E2 envelope glycoprotein of HGV, there is no much data on its molecular and immunological features, clinical significance and its pathogenic potential in hepatitis or any other GBV-C related diseases. So, results of the present study may explain some structural, physiological and immunological functions of this protein in GBV-C, as well as designing new diagnostic kits and besides, help to better understandingE2 protein characteristic and other members of Flavivirus family, especially HCV.

## 1. Introduction

In 1995 and 1996, different isolates of the same new enveloped, RNA positive-stranded flavivirus-like particles with a genomic size of about 9.3 Kb, were isolated by two independent research groups, which named GB virus C (GBV-C) and hepatitis G virus (HGV), respectively. This RNA contains an open reading frame (ORF) which encodes polyprotein with about 2900 amino acids length. By viral/host proteases the polyprotein of GB virus C is cleaved into structural proteins (include; Core, E1 and E2) and nonstructural proteins (include; NS2, NS3, NS4, NS5a and NS5b) (1, 2). Until now, 6 genotypes were reported in different geographical regions of the world (3). This virus could transmit parentally through different routes (1, 4) and is common in some parts of the world such as Iran (5). Overview of HGV infection in Iranian different population revealed that HGV coinfection is highly prevalent among patients and blood donors infected with HIV or HCV, and negative HIV, HCV and HBV populations are a low risk group for HGV infection. There is intermediate frequency among patients on hemodialysis, and those with thalassemia, IVDUs, and leukemia (5, 6). Occupational infection offers the lowest rates, and does not need to monitor blood donors before transfusion (5). 

There are evidences on reducing HCV-related liver morbidity associated with GB virus C (GBV-C) and inhibitory effect of GB virus C on HCV/HIV viremia, survival, a lower mortality rate, slower disease progression in patients with coinfection and also, GBV-C could play role as a predictor for hospital acquired infection (7, 8). Interferon-alpha treatment caused a marked but usually transient reduction in serum GBV-C/HGV RNA, and ribavirin had, at most, a modest antiviral effect (9). 

E2 envelope protein of GB virus C plays role in virus entry into the cytosol, genotyping (10), the ideal targets for vaccine development, and a marker to diagnose GBV-C infections (11), and besides, the concomitance between E2 protein and gp41 protein of HIV-1 affects protein folding and whether it forms a non active complex with gp41-FP. In primates (Chimpanzees model in HCV) it has been reported that purified recombinant envelope glycoproteins (E1 and E2) had potential to protect against challenge with homologous virus, therefore these proteins are the ideal targets for vaccine development (11).

Nowadays, viral-related bioinformatics analysis tools are powerful approaches to predict molecular features such as similarity, glycosylation/phosphorylation/ Palmitoylation sites, epitope recognition, protein primary secondary/ tertiary structures of proteins encoded in viral genomes (12).

One of the branches of bioinformatics is Immunoinformatics or computational immunology which has emerged recently as an important field in the analysis, immune function modeling and prediction of both B and T cell epitopes, novel vaccines designing and allergenicity analysis (13, 14).

Glycoprotein glycosylation characteristics are known to be in association with changes of virulence, cellular tropism in enzymes, and survival of viruses (15). Palmitoylation is an important lipid modification (16), which enhances the protein surface hydrophobicity, membrane affinity and aggregation, modulating proteins' membrane trafficking, stability, and cell signaling (17, 18).Protein phosphorylation has role in regulating physiological functions of virus proteins in replication and assembly processes (19). 

Different structure prediction approaches with different reliability simplify the discovery process in biology, and provide a structural framework for new hypotheses. They were also continuously developed and evaluated (20, 21). Understandings of a protein structure provide deep insight into its interaction with other proteins and small molecules. On the other hand, protein interactions define the protein function, and its biological role in an organism. So, protein structures and structural features prediction is a fundamental area of computational biology (22). To date, there is no data on computational molecular features and Immunoinformatics study of GB virus C E2 protein; although, there are a lot of reports about HCV E2 protein analysis (23-28). 

The purposes of our study are to multi aspect molecular evaluation of GB virus C E2 protein from its characteristics, mutations, structures and antigenicity. These valuable information would help to new directions for future research such as designing diagnostic kits and help to better understanding similarities or differences of biological features of GB C virus in comparison with other members of the Flavivirus family, especially Hepatitis C virus (HCV). The interplay between experimental and computational biology has enormous benefits and providing invaluable Information in many different areas of the sciences.

## 2. Evidence Acquisition

### 2.1. Retrieving Reference Sequences of E2 Protein

Complete putative E2 (Accession number (AC)NP_803203) of GB virus C/Hepatitis G virus mentioned as a reference sequence in National Center for Biotechnology Information (NCBI) Databases (http://ncbi.nlm.nih.gov/) was retrieved. In bioinformatics analyzing a reference sequence (RefSeq) is mostly preferred causes that well annotated and nucleotide sequence (DNA, RNA) and its protein products are available and reliable.

### 2.2. Entropy Plot and Alignment for Finding the Mutational/Conservative Regions 

**Figure 1.**
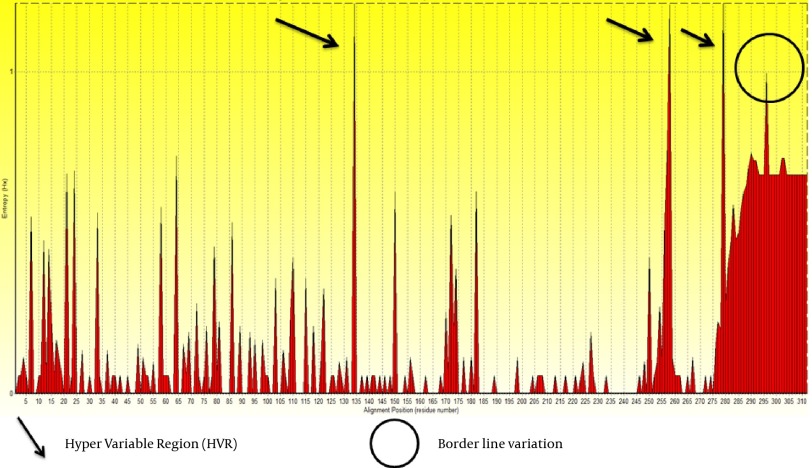
Variation along E2 Protein Sequences of Hepatitis G Virus of GB Virus C Shown by Entropy Plot Regions above threshold 1 were supposed as high variable regions, and arrows represent these positions. Circle shows border line variation that did not include in variation analysis. Entropy Values (Hx) are a measure of variation at each amino acid position in the set of aligned sequences.

We retrieved 100 sequences of E2 protein of GB virus C from NCBI by direct searching. Obtained sequences were aligned, analyzed and trimmed in Bioedit 7.7.9 software. Subsequently, short sequences and areas with ambiguous alignment were excluded. Then, Entropy values (Hx) were measured. This analysis measures variation at each amino acid position in the set of aligned sequences. Results are shown in Figure 1. 

### 2.3. Analyzing Primary Structure of E2 Protein, Amino Acid Distributions, and Transmembrane Topology

The primary protein structure of E2 (e.g. length, Molecular weight (Mw), Isoelectric point (pI) and amino acid distribution) was arranged in Table 1 by utilizing Expasy tools (http://web.expasy.org/protparam/). For amino acid distribution evaluation we used lrrfinder server (http://www.lrrfinder.com/lrrfinder.php). Finally, transmembrane topology of E2 protein was checked by using TMHMM server ( 29 ). 

**Table 1.** Parameters Computed Using Expasy Prot Param Tool

Accession Number (AC.)	E2 (AC. NP_803203)
**No. of amino acids**	312
**Mol. Wt ** ^**a**^	33947.8
**pI ** ^**a**^	8.69
**Total -R and +R ** ^**a**^	21, 26
**Inst.II** ^** a**^	44.95
**AI, GRAVY ** ^**a**^	100.58, 0.333

^a^ Abbreviations: Mol. Wt, Molecular Weight; pI, Theoretical Isoelectric Point; -R, Number of negative charged residues (Arg + Lys); +R, Number of positive charged residues(Asp + Glu); EC, Extinction Coefficient at 280 nm; II, Instability Index; AI, Aliphatic Index; GRAVY, Grand Average Hydropathicity.

### 2.4. Analysis of N-glycosylation, Potential Phosphorylation and Palmitoylation Sites

We used NetNGlyc 1.0 server (http://www.cbs.dtu.dk/services/NetNGlyc) and NetPhos 2.0 server (http://www.cbs.dtu.dk/services/NetPhos.) to predict N-Glycosylation and Phosphorylation sites in E2 protein. These two servers are both taking advantage of artificial neural networks (ANN) for this prediction. NetNGlyc 1.0 server examines the sequence context of Asn-Xaa-Ser/Thr sequences and the NetPhos 2.0 server predicts serine, threonine and tyrosine phosphorylation sites. Palmitoylation sites were predicted with the medium threshold frequency by using services at http://csspalm.biocuckoo.org/prediction.php, in particular CSS-Palm 2.0 software.

### 2.5. Prediction of Secondary Structure of E2 Protein

The secondary structure of the protein was evaluated by using bioinformatics tools available on the website; http://npsa-pbil.ibcp.fr. The method of GOR4 (http://npsa-pbil.ibcp.fr/cgi-bin/npsa_automat.pl?page=npsa_ gor4.html) was used to identify the alpha helices, beta strands, and coil residues. 

### 2.6. Prediction of Tertiary Structure of E2 Protein

As we could not find any matches in SWISS-PROT for E2 to analyze functional and structural motifs, we used SCRATCH suite (http://www.igb.uci.edu/) combines machine learning methods, evolutionary information, fragment libraries and energy functions to predict protein structural features and tertiary structures. The 3D model is visualized by the Swiss-Pdb Viewer software.

### 2.7. Prediction of T-cell and B-cell Epitopes 

#### 2.7.1. Prediction of T-cell Epitopes

IEDB (Immuno Epitope Database) server website (http://tools.immuneepitope.org/mhci/) provides access to predictions of peptide binding to MHC class I molecules.

It estimates IC50 values for peptides binding to specific MHC molecules. List box for selecting the prediction method allows to use different MHC class I binding prediction methods such as Artificial Neural Networks (ANN), Stabilized Matrix Method (SMM), SMM with a Peptide MHC Binding Energy Covariance matrix (SMMPMBEC), Scoring Matrices derived from Combinatorial Peptide Libraries (Comblib_Sidney2008), Consensus method (e.g. ANN, SMM, and CombLib), and NetMHCpan. 

HLA-A*0201 is the most frequent allele and also the first human HLA allele for which peptide binding prediction was developed (30). Therefore, predictions of epitopes were checked for this allele.

#### 2.7.2. Prediction of B-cell Epitopes

#### 2.7.2.1 Prediction of Linear B-cell Epitope Based on Physico-Chemical Profiles

E2 protein antigenicity prediction was checked based on hydrophobicity, assessment of solvent accessibility regions, flexibility, secondary structure (Beta-Turn prediction), and Kolaskar and Tongaonkar method (31). Kolaskar and Tongaonkar prediction method needs more attention, as is based on a semi empirical approach, developed on physic-chemical properties of amino acid residues (i.e. hydrophilicity, accessibility and flexibility). This approach has the efficiency to detect antigenic peptides with about 75% accuracy. To achieve these goals we exploit Bcepred server (32). The accuracy of prediction in this server models varies from 52.92% to 57.53% based on various properties. The highest accuracy obtained for this server was 58.70% at threshold 2.38 when it combined four amino acid profiles (hydrophilicity, flexibility, polarity and exposed surface).

##### 2.7.2.2. B-cell Epitope Prediction by Machine Learning Approaches

Several methods using machine learning approaches have been introduced. The hybrid method applied in this study is composed of hidden Markov model, Feed forward and recurrent neural network, subsequence kernel based SVM and SVM which are used in BepiPred (33), ABCPred (34), BCPred (35) and ABCPred, respectively.

### 2.8. Comparative Analysis of Consensus Epitope for B-cell, Visualization of Epitopes and Variations in 3D Structure

Finally, we compared all the analyses mentioned above to interpret unique molecular features and Immunoinformatics of this protein. Also, the predicted B-cell epitopes were evaluated whether they were present in outer transmembrane regions, using TMHMM results. Epitopes exposed on the surface of the membrane were selected and subjected to further analysis. Moreover, variations represented in entropy plot were checked in 3D model.

### 2.9. Homology Models Validation

The quality evaluation of the modeled structure is an essential step in homology modeling. The geometric estimation of the modeled 3D structure (tertiary structure) was performed using the Ramachandran plot (http://mordred.bioc.cam.ac.uk/~rapper/rampage.php). Ramachandran plots is The two-dimensional (2D) scatter plots of φ, ψ (or torsional angles) which tests whether the model structure is stereo-chemically stable and the number of outliers (36). The plot included three regions; the favored, allowed and outlier regions.

## 3. Results

### 3.1. Entropy Plot for Finding the Mutational and Conservative Sites

Based on the entropy plot, 3 hyper variable regions (HVR) observed along E2 protein that located in residues 133-135, 256-260 and 279-281. HVR are regions in sequence with highest variation in different isolates of virus. Besides, highest conservation observed at amino acids 152-168 and 183-248. Residue 256-260 is located in outer membrane region of E2 protein (see 4.2.), and this variability may help GB virus C to escape immune response of its host.

### 3.2. Analyzing Primary Structure, Amino Acid Distribution of E2 Protein and Transmembrane Topology

Summarized obtained data from Expasy ProtParam tool presented in Table 1. 

An average length of protein sequence and molecular weight of constructs were mentioned in the Table 1. Isoelectric point (pI) is the pH point in which the protein surface is covered with charge, but net charge of protein is zero. Isoelectric point (pI) is important to estimate solubility, and the mobility in an electric field is zero. The calculated isoelectric points (pI) were 8.69 for this protein. The computed value more than 7 indicates that the E2 protein has basic nature. The instability index provides the estimation of the stability of protein in in-vitro. This protein is classified as unstable regarding instability index. The high aliphatic index (100.58) reflects that E2 protein is stable for a variety of temperature ranges. The Grand Average Hydropathicity (GRAVY) values had positive results (0.333), which indicates the low hydrophilicity of protein and low interaction of the protein with surrounding water molecules. 

In physicochemical analysis, it was revealed that the most abundant amino acid residues were glutamic and glycine. 

**Figure 2.**
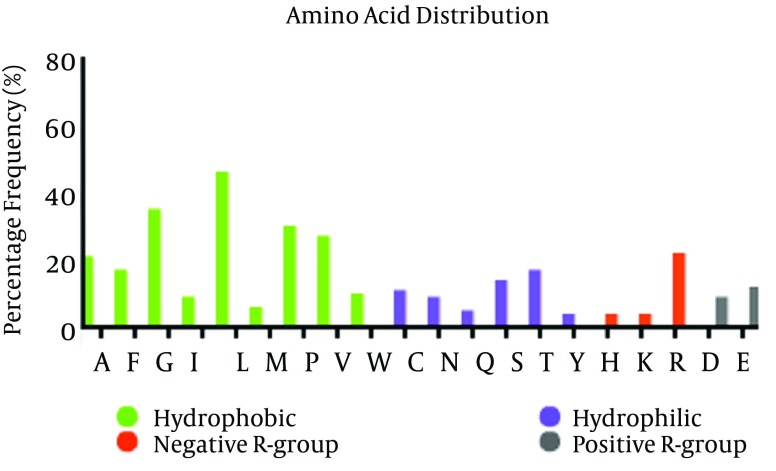
Amino acid Distribution and Composition Frequency of each amino acid, rate of hydrophobic/Hydrophilic residues and positive and negative R-Group in E2 Protein are depicted in figure. This Figure 2 shows that hydrophobic residues are significantly more frequent, as it reflects the hydrophobicity nature of most parts of E2 protein.

Distribution of amino acid frequency in E2 protein showed that hydrophobic residues are more frequent than hydrophilic residues, and also, negative R-group to positive R-group (Figure 2). So, most part of this protein is hydrophobic and locates in membrane. 

**Figure 3.**
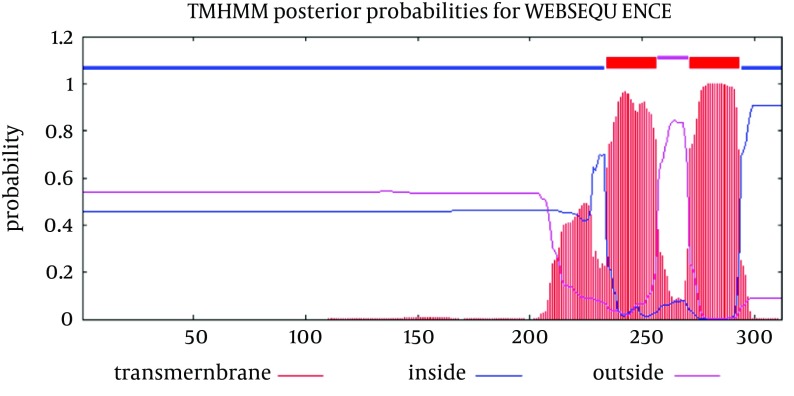
Transmembrane Topology of E2 Protein Red color: Transmembrane region of E2 protein, Blue color: Inside regions and violet color: Outside membrane regions. Vertical axis and horizontal axis are probability of prediction (transmembrane, inside or outside) and order of amino acids in protein sequence of E2 protein, respectively.

Analysis of transmembrane topology using the TMHMM online server found that residues 257-270 presented outside while residues 234- 256 and 271-293 were transmembrane region, and residues 1- 233 and 294-312 were inside the core region of the protein (Figure 3). Also, this analysis would help to select efficient and effective B-cell epitopes. 

### 3.3. Analyzing Potential Glycosylation, Phosphorylation and Palmitoylation Sites 

**Figure 4.**
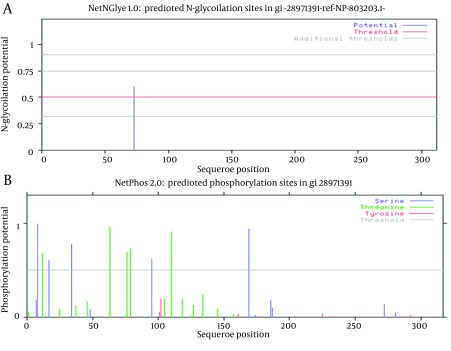
Representation of Predicted Glycosylation and Phosphorylation Sites A shows glycosylation, and B represents phosphorylation. Details of each plot are arranged in Table 2.

Just one N-glycosylation site (residue 73) was found in E2 protein of GB virus C (Figure 4 and Table 2). Potential phosphorylation sites analysis revealed that there were 5 Serine and Threonine potential phosphorylated peptides in the E2 protein (Table 2). Details of phosphorylation analysis were depicted in Figure 4. We found both of glycosylation and phosphorylation sites located inside of the membrane region of E2 protein. 

**Table 2.** Details of Glycosylation and Phosphorylation Sites

Envelope Glycoprotein	E2 (AC NP_803203)
**Glycosylation positions and related sequence**	73 (NRTT)
**Phosphorylation positions**	5 Serine Phos. Sites (include; 8, 17, 34, 95, 169), 5 Threonine Phos.sites(include; 12, 63, 76, 79 , 110), 0 Tyrosine Phos. site

To account for the possible Palmitoylation sites we applied CSS-PALM 3.0 software by choosing medium threshold (Table 3). Results showed two palmitoylation sites in this protein which are near each other. Palmitoylation sites are located inside of this protein regarding TMHMM online server. 

**Table 3.** Details of Palmitoylation Sites Prediction

Position	Peptide	Score	Cutoff
**38**	RPASCGTCVRDCWPE	0.417	0.408
**42**	CGTCVRDCWPETGSV	0.435	0.408

### 3.4. Protein Secondary Structure Prediction

**Figure 5.**
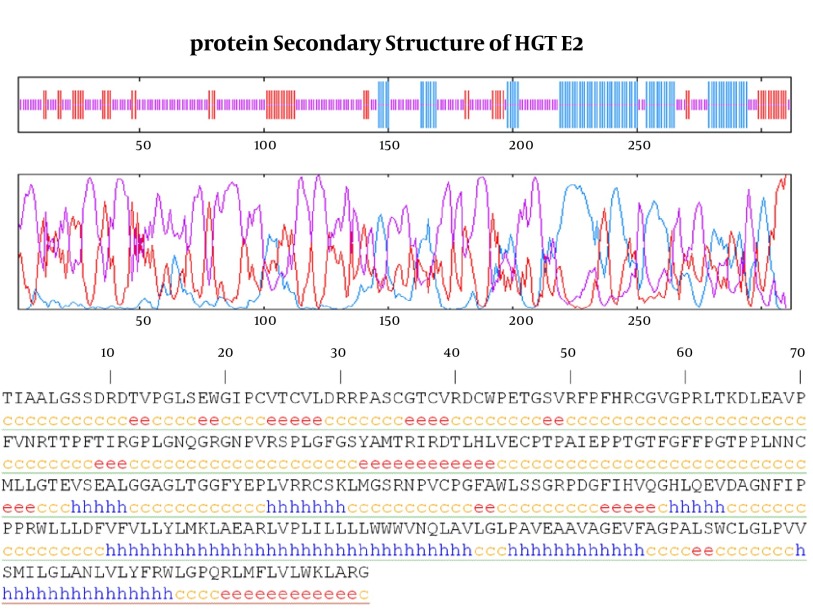
GOR IV Secondary Structure Prediction Method Graphic visualizes the prediction. Blue; Alpha Helix (α-helix), Red; Extended Strand (β-strand), Violet; Other states (Coils).

As it shown in Figure 5, six α-helix, 12 β-strandexist in E2 protein of GB virus C. 

Finally calculating Coils (Beta turns) revealed 17 coil region in E2 structure. Outer membrane region predicted by TMHMM online server has α-helix (dominant structure), small β-strand as well as coil structure.Transmembrane regions have α-helix predominantly.

### 3.5. Prediction T-cell and B-cell Epitopes

#### 3.5.1. Prediction T-cell Epitopes

The predicted epitopes were evaluated for their immunogenicity, and epitopes found to be immunogen in nature were introduced as major immunogenic epitopes for T CD8+-cell (Table 3). Epitope NH3-LLLDFVFVL-COOH (Rank 0.2), NH3-ILLLLWWWV-COOH (0.3), NH3-LMFLVLWKL-COOH (0.4), and NH3-KLMGSRNPV-COOH (0.5) at positions 215-223, 238-246, 301-309 and 170-178 respectively, were found to have the highest antigenicity among all epitopes. Also, none of the predicted epitopes were located in HVR regions. 

**Table 4.** Predicted T CD8+ cell Epitopes by IEDB Server,forSpecificity Reasons Only Epitopes Under Rank 2 Were Selected, Epitope Lengths Were Fixed on 9mer

Protein	Allele	Start- End	Sequence	Method used	Rank
**E2**	HLA-A*02:01	215-223	LLLDFVFVL	Consensus (ann,smm,comblib_sidney2008)	0.2
**E2**	HLA-A*02:01	238-246	ILLLLWWWV	Consensus (ann,smm,comblib_sidney2008)	0.3
**E2**	HLA-A*02:01	301-309	LMFLVLWKL	Consensus (ann,smm,comblib_sidney2008)	0.4
**E2**	HLA-A*02:01	170-178	KLMGSRNPV	Consensus (ann,smm,comblib_sidney2008)	0.5
**E2**	HLA-A*02:01	214-222	WLLLDFVFV	Consensus (ann,smm,comblib_sidney2008)	0.6
**E2**	HLA-A*02:01	241-249	LLWWWVNQL	Consensus (ann,smm,comblib_sidney2008)	0.6
**E2**	HLA-A*02:01	233-241	RLVPLILLL	Consensus (ann,smm,comblib_sidney2008)	1
**E2**	HLA-A*02:01	221-229	FVLLYLMKL	Consensus (ann,smm,comblib_sidney2008)	1.1
**E2**	HLA-A*02:01	281-289	SMILGLANL	Consensus (ann,smm,comblib_sidney2008)	1.3
**E2**	HLA-A*02:01	282-290	MILGLANLV	Consensus (ann,smm,comblib_sidney2008)	1.3
**E2**	HLA-A*02:01	216-224	LLDFVFVLL	Consensus (ann,smm,comblib_sidney2008)	1.5
**E2**	HLA-A*02:01	112-120	HLVECPTPA	Consensus (ann,smm,comblib_sidney2008)	1.6
**E2**	HLA-A*02:01	219-227	FVFVLLYLM	Consensus (ann,smm,comblib_sidney2008)	1.6
**E2**	HLA-A*02:01	253-261	GLPAVEAAV	Consensus (ann,smm,comblib_sidney2008)	1.9

#### 3.5.2. Prediction B-Cell Epitopes of E2 Protein

#### 3.5.2.1 Prediction of Linear B-Cell Epitopes Basedon Physic-Chemical Properties 

**Figure 6.**
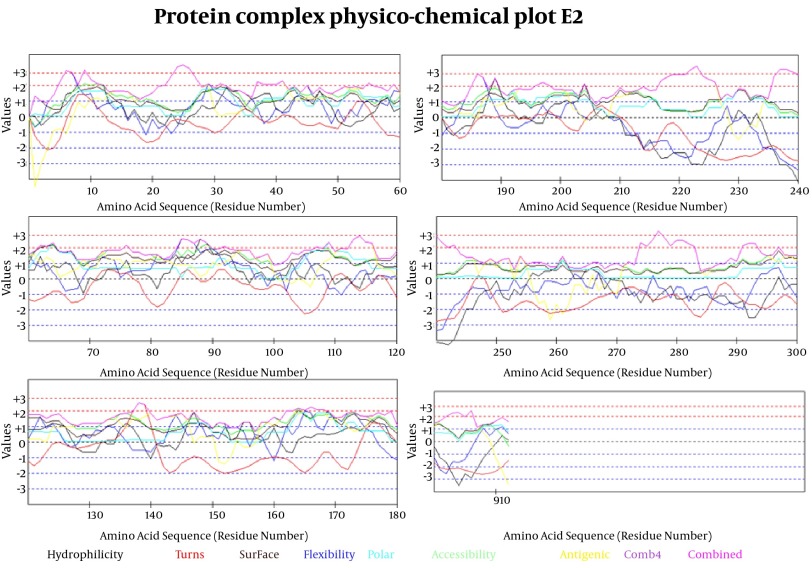
Selected Profiles Were Hydro, Flexi, Access, Turns, Surface, Polar and Antigenic and Respective Thresholds Were 1.9, 2, 1.9, 2.4, 2.3, 1.8 and 1.9. Combination of properties (Comb4).

In Figure 6 we evaluated the existence of linear B-cell epitopes in E2 protein sequence based on physico-chemical properties. Details of these predictions are arranged in Table 4. 

**Table 5.** Prediction of B-cell Epitopes Using Any of the Physico-Chemical Properties; Hydrophilicity, Flexibility/Mobility, Accessibility, Polarity, Exposed Surface and Turns

Profiles	Positions in E2 protein Sequence
**Hydrophilicity**	1MGPPSSAAACSRGSPRILRVRAGGISFFYTIMAVLLLLLVVEAGAILAPATHACRANGQYFLTNCCAPEDIGFCLEGGCLVALGCTICTDQCWPLYQAGLAVRPGKSAAQLVGELGSLYGPLSVSAYVAGILGLGEVYSGVLTVGVALTRRVYPVPNLTCAVACELKWESEFWRWTEQLASNYWILEYLWKVPFDFWRGVISLTPLLVCVAALLLLEQRIVMVFLLVTMAGMSQGAPASVLGSRPFDYGLTWQTCSCRANGSRFSTGEKVWDRGNVTLQCDCPNGPWVWLPAFCQAIGWGDPITYWSHGQNQWPLSCPQYVYGSATVTCVWGSASWFASTSGRDSKIDVWSLVPVGSATC360
**Flexibility**	1MGPPSSAAACSRGSPRILRVRAGGISFFYTIMAVLLLLLVVEAGAILAPATHACRANGQYFLTNCCAPEDIGFCLEGGCLVALGCTICTDQCWPLYQAGLAVRPGKSAAQLVGELGSLYGPLSVSAYVAGILGLGEVYSGVLTVGVALTRRVYPVPNLTCAVACELKWESEFWRWTEQLASNYWILEYLWKVPFDFWRGVISLTPLLVCVAALLLLEQRIVMVFLLVTMAGMSQGAPASVLGSRPFDYGLTWQTCSCRANGSRFSTGEKVWDRGNVTLQCDCPNGPWVWLPAFCQAIGWGDPITYWSHGQNQWPLSCPQYVYGSATVTCVWGSASWFASTSGRDSKIDVWSLVPVGSATC
**Accessibility**	1MGPPSSAAACSRGSPRILRVRAGGISFFYTIMAVLLLLLVVEAGAILAPATHACRANGQYFLTNCCAPEDIGFCLEGGCLVALGCTICTDQCWPLYQAGLAVRPGKSAAQLVGELGSLYGPLSVSAYVAGILGLGEVYSGVLTVGVALTRRVYPVPNLTCAVACELKWESEFWRWTEQLASNYWILEYLWKVPFDFWRGVISLTPLLVCVAALLLLEQRIVMVFLLVTMAGMSQGAPASVLGSRPFDYGLTWQTCSCRANGSRFSTGEKVWDRGNVTLQCDCPNGPWVWLPAFCQAIGWGDPITYWSHGQNQWPLSCPQYVYGSATVTCVWGSASWFASTSGRDSKIDVWSLVPVGSATC
**Turns**	Nothing
**Exposed Surface**	Nothing
**Polarity**	1MGPPSSAAACSRGSPRILRVRAGGISFFYTIMAVLLLLLVVEAGAILAPATHACRANGQYFLTNCCAPEDIGFCLEGGCLVALGCTICTDQCWPLYQAGLAVRPGKSAAQLVGELGSLYGPLSVSAYVAGILGLGEVYSGVLTVGVALTRRVYPVPNLTCAVACELKWESEFWRWTEQLASNYWILEYLWKVPFDFWRGVISLTPLLVCVAALLLLEQRIVMVFLLVTMAGMSQGAPASVLGSRPFDYGLTWQTCSCRANGSRFSTGEKVWDRGNVTLQCDCPNGPWVWLPAFCQAIGWGDPITYWSHGQNQWPLSCPQYVYGSATVTCVWGSASWFASTSGRDSKIDVWSLVPVGSATC
**Antigenic Propensity**	1MGPPSSAAACSRGSPRILRVRAGGISFFYTIMAVLLLLLVVEAGAILAPATHACRANGQYFLTNCCAPEDIGFCLEGGCLVALGCTICTDQCWPLYQAGLAVRPGKSAAQLVGELGSLYGPLSVSAYVAGILGLGEVYSGVLTVGVALTRRVYPVPNLTCAVACELKWESEFWRWTEQLASNYWILEYLWKVPFDFWRGVISLTPLLVCVAALLLLEQRIVMVFLLVTMAGMSQGAPASVLGSRPFDYGLTWQTCSCRANGSRFSTGEKVWDRGNVTLQCDCPNGPWVWLPAFCQAIGWGDPITYWSHGQNQWPLSCPQYVYGSATVTCVWGSASWFASTSGRDSKIDVWSLVPVGSATC

**Figure 7.**
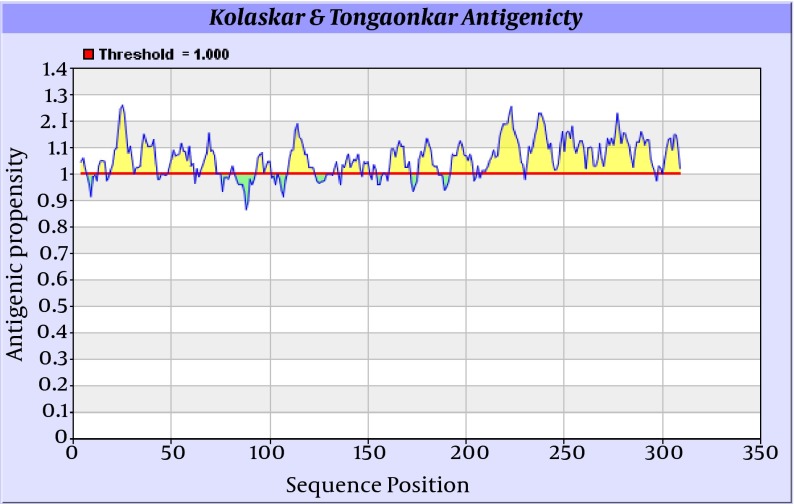
Antigenicity Prediction Plot of E2 Protein by Using Kolaskar-Tongaonkar Algorithm Regions with antigenic propensity scale upper 1 are antigenic regions. Threshold, average, maximum and minimum antigenicity were 1.000, 1.058, 1.259, and 0.866 respectively. Window size and center position were 7 and 4, respectively.

Antigenicity (immunogenicity) prediction plot of E2 (Figure 7) protein revealed span of highly antigenic region that located in residue 231- 296 (fragment of NH3-EARLVPLILLLLWWWVNQLAVLGLPAVEAAVAGEVFAGPALSWCLGLPVVSMILGLANLVLYFRWL-COOH). Also, regions 19-42 (NH3- WGIPCVTCVLDRRPASCGTCVRDC-COOH) and 109-122 (NH3-DTLHLVECPTPAIE-COOH) are other important antigenic regions in this protein. 

##### 3.5.2.2. Prediction Epitopes Based on Machine Learning Approaches

B-cell epitope prediction based on machine learning approaches were performed using

BCPRED server, where criteria were set to have 75% specificity and ABCpred 65.93% accuracy with fixed length of 20 and 16 amino acids (Table 5).Higher score of the peptide means the higher probability as an epitope. 

**Table 6.** Prediction Epitopes Based on Machine Learning Approaches ^a^

">Server	Classifier Specificity	Use Overlap Filter	Epitopes	Scores
**BCPREDS 1.0**	80%	yes	AA_230-250 _(AGMSQGAPASVLGSRPFDYG), AA_296-316 _(AIGWGDPITYWSHGQNQWPL),AA_339-359_ (STSGRDSKIDVWSLVPVGSA)and AA_165-185 _(ELKWESEFWRWTEQLASNYW)	0.977, 0.966, 0.935, 0.887
**ABCpred**	85%	yes	AA_43-59_(AGAILAPATHACRANG), AA_237-253_(PASVLGSRPFDYGLTW), AA_299-215_(WGDPITYWSHGQNQWP), AA_147-163_(ALTRRVYPVPNLTCAV), AA_68-84_(PEDIGFCLEGGCLVAL) and AA_320-236_(YVYGSATVTCVWGSAS)	0.95, 0.93, 0.92, 0.90, 0.85, 0.85

^a^The Predicted B cell Epitopes are Ranked According to Their Score Obtained by Trained Machine Learning Algorithm. All the Peptides Shown Here AreAbove the Threshold Value Chosen. We Tried to Select Highest Score Hits.

### 3.6. Comparative Analysis for Consensus Epitopes for B-cell and 3D Structure of E2 Protein

**Figure 8.**
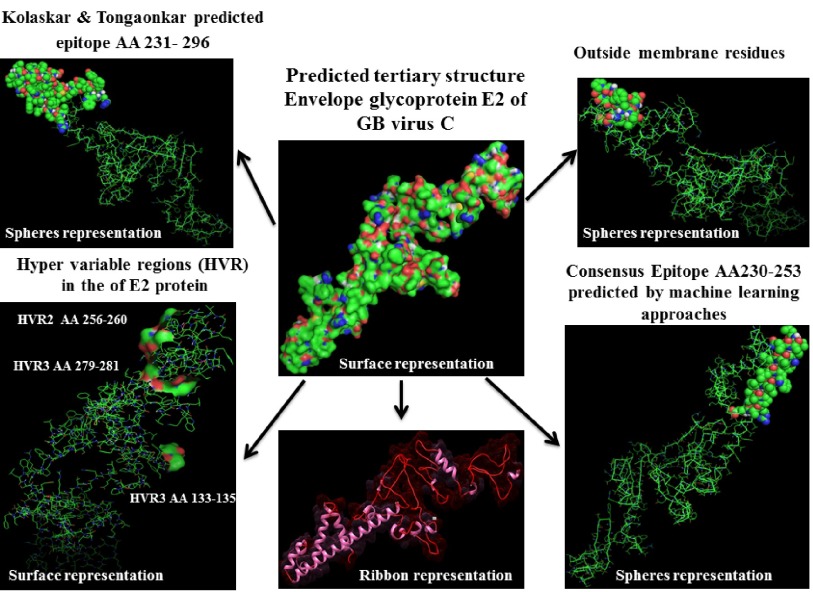
Predicted 3D Structure of the E2 Protein and Visualization of Epitopes and Variations Regions Epitopes predicted by different methods and outer membrane region were shown by spheres representation in 3D Structure. Hyper Variable Regions (HVR) represent by surface representation.

Prediction of B-cell epitopes regarding transmembrane topology (especially outer membrane region), based on physico-chemical properties and machine learning approaches showed that this protein has different regions with potential of immunogenicity, but machine learning method by BCPREDS (specificity 80%) and ABCpred specificity (85%) could not predict epitopes in range of 257-270 (outer membrane region of protein). These servers had a consensus epitope in approximate region of 230-253 that is in transmembrane region based on TMHMM server prediction. In physico-chemical approaches the best performance was seen by Kolaskar-Tongaonkar algorithm in which a part of epitope Residue 231- 296 (fragment of NH2- EARLVPLILLLLWWWVNQLAVLGLPAVEAAVAGEVFAGPALSWCLGLPVVSMILGLANLVLYFRWL-COOH) was located in outer and transmembrane of E2 protein (Figure 8). These epitopes are optimal for immunization and diagnostic programs. 

### 3.7. Validation Modeled Structure by Ramachandran Plot Assessment

**Figure 9.**
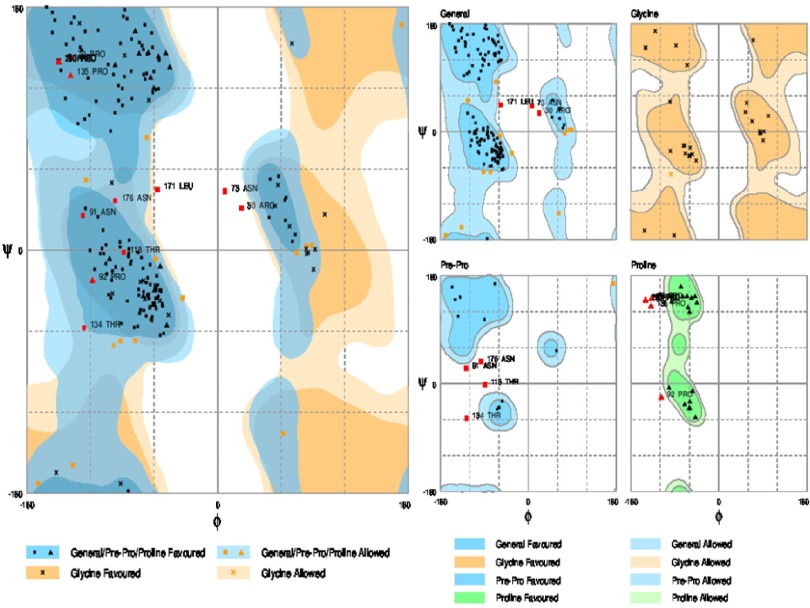
Ramachandran Plot of Predicted Model for the E2 Protein of Hepatitis G Virus RAMPAGE Server Considers Torsional Angles ψ Against φ of Amino Acid Residues in Protein Structure and Results Defining in Favored, Allowed and Outlier Categories.

3D model of the E2 protein with a total number of 310 amino acids was validated using the Ramachandran plot. Assessment of the plot (Figure 9) revealed that 90.4% of residues (281 amino acids) are in the favored regions, 4.5% residues (26 amino acids) in allowed regions and 4.8% residues (15 amino acids) are in the outlier region. The overall percentage of residues in favored and allowed region was 94.9. Therefore, the modeled structure is suitable. 

## 5. Discussion

Here we provided deep insight into the computational molecular features and Immunoinformatics characteristic of E2 protein of GBV-C/HGV by using various bioinformatics techniques.

GBV-C and HGV are closely related isolates of the same virus, with more than 95 percent sequence homology (37). GBV-C and HGV are reported to have a mutation rate lower than the 1.4-1.9 × 10-3 base substitutions per site per year reported for HCV (38, 39).

RNA virus genomes (due to the lack of proofreading ability of their RNA-dependent RNA polymerase) have special potential to undergo mutation at high frequencies, and under selective pressures rapidly generate populations of viral variants. Such variability helps to evading of virus from clearance by both T- and B-cell immunity (40).

Three different HVR (HVR1133-135, HVR2256-260 and HVR3279-281) observed along E2 protein. Besides, residue HVR2256-260 located in outer membrane region of E2 protein. Different researchers suggest that HCV hypervariable region 1 (HVR1) is located in a spanning of 27–31 (or 25-30 in some reports) residues at E2 glycoprotein which is the main target of the anti-HCV neutralizing response and hence plays an important role in providing viral persistence (41, 42). Substitutions of amino acid in HVR1 during HCV infection provide groups of genetically related variants named quasi species (43), that some of these mutants have potential to escape immune response and persist after sero-conversion (42). Much of HCV variability is concentrated in the HVR1 region, therefore for designing more successful vaccine it is needed to induce a broad spectrum, and more cross-reactive response against many HVR1 simultaneously, which bioinformatics could achieve this goal (44).

Sequence analysis of the transmembrane topology of HCV E2 in details and its importance are widely discussed (45). These studies revealed that mutations rarely occur at transmembrane sites and there are high conservation, although there is variation in outer membrane region (these conservation of residues are crucial for the viral specific functions) (45-47). In our study, analysis of transmembrane topology using the TMHMM online server for GBV-C envelope E2 revealed that residues 257-270 presented outside while residues 234- 256 and 271-293 were transmembrane regions.

Finding modifications sites, patterns and number of important viral protein such as; N-glycosylation, palmitoylation, phosphorylation etc. have an enormous effects on foldings, entry functions, viral transportation/replication/assembly, infectivity, pathogenicity, immunogenicity as well as it may explain different virulence between different isolates of a virus and also viral genus (48).

In residue 73, N-glycosylation site was found in E2 protein of GB virus C. In case of HCV the ectodomain of envelope glycoproteins E2 has high modification by N-linked glycans and defined 11 potential glycosylation sites (49, 50), that E2 glycosylation sites show conservation. Indeed, comprehensive sequence analyses of potential glycosylation sites in E2 indicate that 9 of the 11 sites are strongly conserved (49, 50). In this research, phosphorylation sites analysis revealed that there were 5 Serine/Threonine potential phosphorylated peptides. Both of glycosylation and phosphorylation sites were located inside of the membrane region of E2 protein. 

Also, there are reports on in-silico evaluation of glycosylation, phosphorylation and palmitoylation in other viral proteins such as S1 protein from Infectious Bronchitis Virus (IBV), and they finally interpreted that there is differences in number and location of mentioned properties between isolates but most of the glycosylation, phosphorylation and Palmitoylation sites were conserved within specific genotypes (51). These conserved residues are crucial for the viral specific functions. Also, our results showed positions 38 and 42 palmitoylated in E2 protein of GB virus C. Several studies reported evaluation of palmitoylation sites in influenza virus, HIV-1, Semliki Forest virus and Infectious Bronchitis Virus (51), and revealed impact of palmitoylation on viral biology and functions. 

Structure prediction approaches have been continuously developed and they greatly accelerated and simplified discovery of biological features of macromolecule and provided a structural framework for novel and innovative hypotheses. It might notice that different methods have different reliability, and this subject has to be taken into account while using their results and compare the prediction with an experimental result (21). Six α-helix, 12 β-strand and 17 Coils structure were present in E2 protein of GB virus C. Outer membrane region has α-helix (dominant structure), small β-strand as well as coil structure.Transmembrane regions have α-helix predominantly.

The data extracted from the three-dimensional structure of a protein is essential for understanding and solving the details of its molecular function, and gives valuable knowledge for the development of effective rational strategies for experiments such as findings disease related mutations, site directed mutagenesis, or vaccine and drug design based on protein structure ( 22 ). In this work, we visualized positions of variability and epitopes in 3D structure (Figure 8). 

The predicted epitopes for T CD8+-cell (Table 3) with highest antigenicity (immunogenicity) for E2 protein in this study were AA215-223NH3-LLLDFVFVL-COOH, AA238-246 NH3-ILLLLWWWV-COOH, AA301-309 NH3-LMFLVLWKL-COOH, and AA170-178 NH3-KLMGSRNPV-COOH, respectively. 

By comparative analysis of B-cell epitopes between physico-chemical and machine learning approaches regarding 3D/secondary structure and outer membrane region, the best performance was seen by Kolaskar-Tongaonkar algorithm. This epitope was residue 231- 296 (fragment of NH3-EARLVPLILLLLWWWVNQLAVLGLPAVEAAVAGEVFAGPALSWCLGLPVVSMILGLANLVLYFRWL-COOH) (Figure 8). So, this epitope is optimal for immunization and diagnostic methods. 

The comprehensive analysis of a protein with important roles has never been easy, especially when we attempt to make statements from different aspects about this protein. In case of E2 envelope glycoprotein of HGV, there is no much data on its molecular and immunological features, clinical significance and its pathogenic potential in hepatitis or any other GBV-C related diseases. So, results of the present study may explain some of its structural, physiological and immunological functions in GBV-C virus, as well as help to better understanding E2 protein potential of other members of Flavivirus family, especially HCV.
